# Synergistically strengthened 3D micro-scavenger cage adsorbent for selective removal of radioactive cesium

**DOI:** 10.1038/srep38384

**Published:** 2016-12-05

**Authors:** Sung-Chan Jang, Sung-Min Kang, Yuvaraj Haldorai, Krishnan Giribabu, Go-Woon Lee, Young-Chul Lee, Moon Seop Hyun, Young-Kyu Han, Changhyun Roh, Yun Suk Huh

**Affiliations:** 1 Department of Biological Engineering, Biohybrid Systems Research Center (BSRC), Inha University, 100 Inha-ro, Incheon 22212, Republic of Korea; 2 Biotechnology Research Division, Advanced Radiation Technology Institute (ARTI), Korea Atomic Energy Research Institute (KAERI), 29 Geumgu-gil, Jeongeup-si, Jeonbuk 56212, Republic of Korea; 3 Department of Chemical Engineering, Chungnam National University, 99 Daehak-ro, Daejeon 34134, Republic of Korea; 4 Department of Energy and Materials Engineering, Dongguk University-Seoul, 30 Pildong-ro 1-gil, Seoul 04620, Republic of Korea; 5 Quality Management Team, Korea Institute of Energy Research (KIER), 152 Gajeong-ro, Daejeon 34129, Republic of Korea; 6 Department of BioNano Technology, Gachon University, 1342 Seongnam-daero, Seongnam-si, Gyeonggi-do 13120, Republic of Korea; 7 Measurement & Analysisi Team, National Nanofab Center, 291 Daehak-ro, Daejeon 34141, Republic of Korea; 8 Radiation Biotechnology and Applied Radioisotope Science, University of Science and Technology (UST), 217 Gajeong-ro, Daejeon 34113, Republic of Korea

## Abstract

A novel microporous three-dimensional pomegranate-like micro-scavenger cage (P-MSC) composite has been synthesized by immobilization of iron phyllosilicates clay onto a Prussian blue (PB)/alginate matrix and tested for the removal of radioactive cesium from aqueous solution. Experimental results show that the adsorption capacity increases with increasing the inactive cesium concentration from 1 ppm to 30 ppm, which may be attributed to greater number of adsorption sites and further increase in the inactive cesium concentration has no effect. The P-MSC composite exhibit maximum adsorption capacity of 108.06 mg of inactive cesium per gram of adsorbent. The adsorption isotherm is better fitted to the Freundlich model than the Langmuir model. In addition, kinetics studies show that the adsorption process is consistent with a pseudo second-order model. Furthermore, at equilibrium, the composite has an outstanding adsorption capacity of 99.24% for the radioactive cesium from aqueous solution. This may be ascribed to the fact that the AIP clay played a substantial role in protecting PB release from the P-MSC composite by cross-linking with alginate to improve the mechanical stability. Excellent adsorption capacity, easy separation, and good selectivity make the adsorbent suitable for the removal of radioactive cesium from seawater around nuclear plants and/or after nuclear accidents.

The tsunami that followed the earthquake on March 11, 2011 at the Fukushima Daiichi nuclear plant resulted in releasing a radioactive contaminant into the seawater that contains highly radioactive cesium, which is currently present in a wide area of eastern Japan after the tsunami caused a power outage, a subsequent loss of control, and a cooling system failure^1^,^2^. Soon after the nuclear accident, Japan’s Ministry of the Environment reported readings of radioactive cesium seawater as high as 45.5 million Bq/m^3^. The radioactive cesium radionuclide, which has a half-life of 30 years, is hazardous as it exerts toxic effects *via* emission of beta-particles and strong gamma rays. If humans are exposed to radioactive cesium, it is rapidly distributed throughout all muscle tissue, and severe arrhythmia, heart failure, or sudden death can result from the accumulation of radioactive cesium in cardiac muscle^3^,^4^. Several researchers have suggested using physical adsorption methods to remove radioactive cesium from contaminated water. For example, biopolymers^5^, nanocomposite^6^,^7^, and clay^8^ have all previously been investigated for their capacity to remove radioactive cesium through the interaction between the negatively charged surfaces of naturally occurring adsorbents and the positive charge of radioactive cesium. However, powder-type adsorbents cannot be used to treat contaminated water in a real, open environment because there is no easy way to collect the adsorbents after they are used. In particular, adsorbents can cause blocking phenomena, which could be addressed by encapsulating the adsorbents with suitable modification that could alleviate the clogging and resolve the post-treatment separation problem^9^. Therefore, novel solid absorbents are needed to allow easy separation of the materials from the contaminant environment to prevent secondary contamination.

Prussian blue (PB), and its analogues, is well known for its capacity for selective adsorption of radioactive cesium; hence, it has been recognized as an effective scavenger for radioactive cesium^10^,^11^. However, PB nanoparticles prepared using the precipitation method are usually a very fine powder. In water, PB nanoparticles form a stable colloidal suspension, which makes them difficult to separate from an open environment. Compositing PB with a biopolymer offers a potential solution to this problem.

Alginate has been successfully used as an eco-friendly polymer for the encapsulation of absorbing materials^12^,^13^. Alginate, a salt of alginic acid, is a polysaccharide biopolymer derived from brown algae, and it is composed of β-1, 4-linked D-mannuronic acids, and α-1,4-linked L-guluronic acids arranged in a chain^14^,^15^. It has carboxylic groups, which are the most frequent acidic functional group on the chain, and it has affinities for metal cations^16^. It has attracted much attention in many areas because of its biocompatibility, biodegradability, and rapid gelation ability. Alginate gelation occurs when multivalent cations, such as Ca^2+^, Fe^3+^, Al^3+^, and Zr^4+^, interact with blocks of guluronic residues, diffusing into the sodium alginate solution and replacing Na^+^. This results in the rapid formation of alginate gel^17^. Although alginate hydrogels have been extensively studied for adsorption applications, their use has been restricted by their poor mechanical properties and low adsorption capacities^5^,^18^,^19^. Especially in a marine environment treatment process, the calcium ions are easily detached by chelating agents and monovalent ions, such as Na^+^ and K^+^, that are found in seawater, which results in destabilization of the alginate matrix^20^.

This study aimed to design and synthesize a new microporous three-dimensional (3D) network composite composed of PB encapsulated by alginate beads, which were reinforced by cross-linking aminopropyl-functionalized iron phyllosilicates (AIP) clay, for high adsorption and easy separation of radioactive cesium from contaminated water. The synthesized composite was confirmed by Fourier-transform infrared spectroscopy (FTIR), X-ray diffraction (XRD), X-ray photoelectron spectroscopy (XPS), thermal gravimetric analysis (TGA), and scanning electron microscopy (SEM). The results revealed that the composite adsorbent was highly efficient in the removal of radioactive cesium because of the good ion-exchange property of PB and the excellent adsorption property of the alginate/AIP clay network. Owing to the bio-compatible nature of alginate and AIP clay^5^, they may form a potential class of adsorbents for environmental remediation when fabricated suitably.

## Results and Discussion

### Synthesis of composite

**Figure 1:**
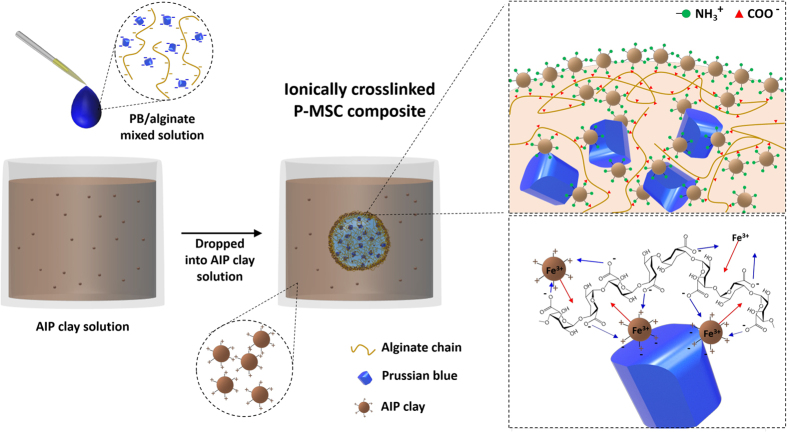
Schematic diagram for the fabrication of a 3D microporous PB/alginate/AIP clay composite.

The schematic diagram for the synthesis of the AIP clay immobilized PB/alginate composite is shown in Fig. 1. The composite was fabricated using a two-step process. In the first step, PB nanoparticles dispersed in alginate solution and AIP clay dispersed in aqueous solution were prepared separately. In the second step, the PB/alginate solution was added drop-wise to the AIP clay solution and the solution underwent gelation for one day to form the 3D microporous PB/alginate/AIP clay composite. Alginate, AIP clay, and PB nanoparticles have different charges, with zeta potential values of −33.34 mV, 41.36 mV, and −33.84 mV, respectively. When PB/alginate is added to the AIP clay solution, the Fe^3+^ ions of the AIP clay (Figure S2) bind to the carboxyl (COO^−^) groups of alginate more strongly than Ca^2+^^21^,^22^. This is because the relative binding affinity of the carboxyl group to metal ion is in the order of Fe^3+^ >> Ca^2+^ >> Na^+^. On the other hand, the amino (-NH_2_) groups of the AIP clay can cross-link with PB and alginate to form a hydrogel *via* ionic interactions^23^. Moreover, the AIP clay acts as an immobilization agent, and it can directly immobilize onto the PB/alginate matrix without forming any intermediate material. This technique allows for the direct solidification of PB/alginate without the need of Ca^2+^. The positive and negative charges of clay and alginate lead to an effective ionic interaction, which creates a porous network. This mechanism is mainly induced by the unique structure and elegant performance of the composite. In comparison to pristine Ca-alginate, the composite combines the intrinsic physical and chemical properties of the organic and inorganic materials, acquires more degrees of freedom to manipulate multiple interactions, creates hierarchical structures, and integrates multiple functionalities^24^,^25^,^26^.

### Structural, morphological, and surface studies

**Figure 2:**
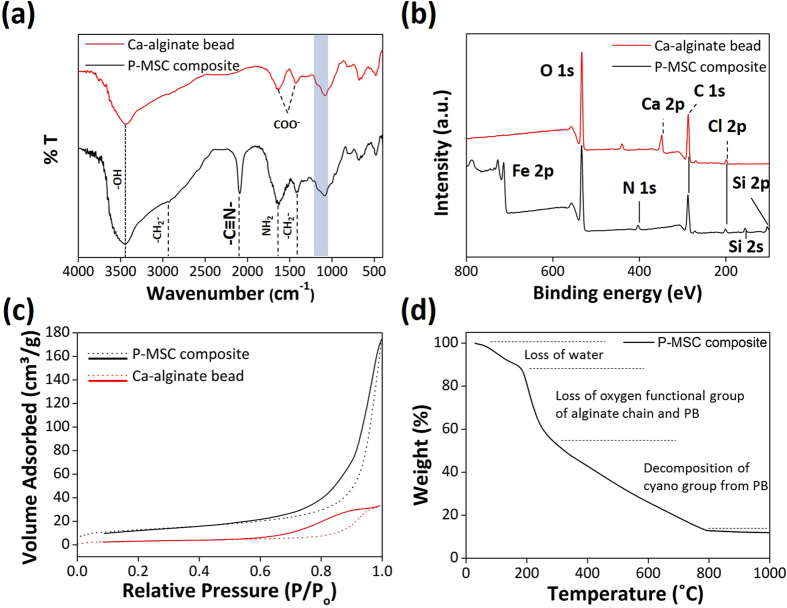
Characterization of Ca-alginate bead and P-MSC composite. (**a**) FT-IR spectra, (**b**) XPS spectra, and (**c**) the BJH N_2_ adsorption/desorption isotherms for Ca-alginate bead and P-MSC composite, and (**d**) TGA curve of the P-MSC composite.

Figure 2a illustrates the FT-IR spectra of Ca-alginate and the P-MSC composite. The FT-IR spectrum of Ca-alginate showed a broad band at 3447 cm^−1^ corresponding to the -OH stretching vibrations, and a small band at 2930 cm^−1^ that was due to the C-H stretching vibrations. The bands at 1637 cm^−1^ and 1430 cm^−1^ were related to the asymmetric and symmetric stretching vibrations of the -COO groups, respectively. The broad band at 1200–1000 cm^−1^ corresponds to the C–O–C stretching vibrations of the carbohydrate rings^27^. For the alginate/AIP clay composite (Figure S1a) and P-MSC composite (Fig. 2a), in addition to the alginate bands, characteristic bands for the AIP clay (-OH stretching at 3444 cm^−1^, -NH_2_ stretching at 1623 cm^−1^, -CH_2_ stretching at 1425 cm^−1^, Si–O–Si stretching at 1035 cm^−1^, and Fe–O stretching at 685 cm^−1^)^28^ were observed. Particularly, the P-MSC composite showed a -C≡N- stretching at 2091 cm^−1^ corresponding to the PB nanoparticles^6^,^29^. These bands provide evidence for the presence of alginate, PB, and AIP clay in the P-MSC composite. Figure 2b shows the survey spectra for Ca-alginate bead and the composite. The sharp peaks in the full scan spectra of Ca-alginate bead revealed the presence of C 1 s (286.58), O 1 s (532.48), and Ca 2p (348.08), whereas the full scan spectra of alginate/AIP clay composite (Figure S1b) and the P-MSC composite showed C 1 s (288.28), O 1 s (534.08), Fe 2p (713.68 eV), Si 2 s (155.08 eV), Si 2p (105.48 eV), and N 1 s (400 eV) peaks, confirming the successful immobilization of the AIP clay over the alginate matrix. The NMR spectroscopic response can be used as a local probe to extract structural information, as the chemical shift of the observed nucleus is very dependent on its environment. ^1^H MAS NMR and ^13^C MAS NMR spectra of Alginate, AIP clay, and P-MSC composite are shown in Figure S1c and S1d. No significant changes in the ^1^H MAS NMR spectra, indicating a certain stability and homogeneity of the obtained materials. The carboxyl group of sodium alginate reacted ionically with the amino group and Fe ion of AIP clay during complexation. However, in ^13^C MAS NMR spectrum of P-MSC composite observed peaks between 50–100 ppm and 150–200 ppm were assigned to PB. The PB analogues are three-dimensional inorganic solids with apparently simple composition (cyanide ligands connecting metal ions) and structure (cubic) cover a large family of (often nonstoichiometric) compounds (Figure S5b). These results confirmed that the AIP clay can cross-linked with PB and alginate to form a hydrogel *via* ionic interactions. The Brunauer–Emmett–Teller (BET) surface area of the composite (Fig. 2c) was calculated to be 44.5 m^2^/g, which was evaluated from the 0.1 < P/P_0_ < 1.0 region of the adsorption curve. The Barrett-Joyner-Halenda pore size distribution suggested the mesoporous nature of the composite, and the average pore diameter and pore volume were observed to be 31.34 nm and 0.3656 cm^3^/g, respectively. Figure 2d illustrates the TGA curve of the composite at a heating rate of 10 °C/min under nitrogen. TGA data showed a mass loss on heating even below 200 °C, probably due to the loss of water. A gradual mass loss in P-MSC composite around 300 °C were observed with an increase in temperature, which could be attributed to the removal of coordinating water from PB and oxygen functional groups from alginate chain. Between 400 and 750 °C, the composite showed a more obvious mass loss, which corresponded to the decomposition of the cyano group^30^,^31^. The degradation over 700 °C was attributed to the combustion of alginate chain. When the temperature increased to 950 °C, reduction in the weight of P-MSC composite was observed due to the pyrolysis of the carbon skeleton. Figure S3 displays XRD patterns of the Ca-alginate bead, Alginate/AIP clay composite, and P-MSC composite. The diffraction peaks of Ca-alginate bead and Alginate/AIP clay composite recorded patterns which confirm their amorphous nature. Generally, the diffraction pattern of the investigated physical mixture was corresponded to the superposition of those of its individual components and revealed that Prussian blue was present, as evidenced by the presence of its diffraction lines. In the XRD spectrum of the composite, all the peaks were assigned to PB. The diffraction peaks were observed at 2θ = 17.4°, 24.8°, 35.3°, and 39.5°, which were indexed to (200), (220), (222), and (400) reflections, respectively, of the face-centered cubic structure of the PB nanoparticles^32^.

**Figure 3:**
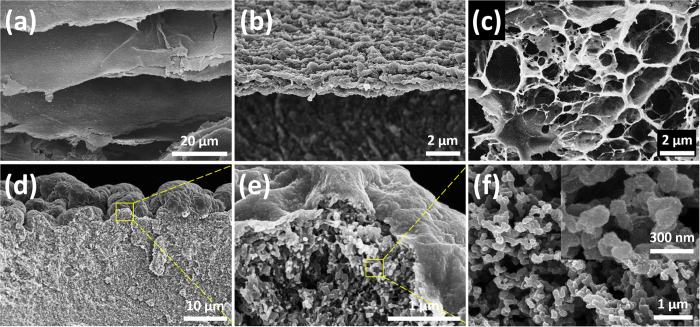
A cryo-fractured cross-section SEM images of (**a**,**b**) Ca-alginate, (**c**) alginate/AIP clay composite, and (**d**) P-MSC composite, (**e**) a magnified image of outer surface and inner structure of the P-MSC composite, and (**f**) close inspection of inner structure of the P-MSC composite.

Figure 3 displays the typical SEM images of Ca-alginate, the alginate/AIP clay composite and the PB/alginate/AIP clay composite. It is clear that the Ca-alginate (Fig. 3a and b) surface was rough and porous (Figure S4a), but it possessed poor mechanical property. However, after alginate was cross-linked with the AIP clay, the material showed a microporous 3D network (Fig. 3c and S4b). Porosity can be correlated to the mechanical performance and adsorption capacity of a matrix; in our case, the adsorption capacity is anticipated for the alginate/AIP clay composite because of the porous structure. The SEM images of PB/alginate/AIP clay composite with different magnifications are shown in Fig. 3d and e. Figure 3d illustrates that the AIP clay nanoparticles were closely packed on the surface of the PB/alginate matrix. Figure 3e showed a pomegranate-like micro-scavenger cage (P-MSC) structure of the composite in which the AIP clay particles were cross-linked with the PB/alginate matrix. Close inspection of the composite (Fig. 3f) clearly showed that the AIP clay nanoparticles, with an average particle size ranging from 30–50 nm (Figure S5a)^33^,^34^,^35^, were immobilized onto the PB/alginate matrix. EDX analysis confirmed the elemental composition of the composites. Owning to the limited depth of EDX (a few microns), EDX result cannot reflect the full information of elemental distributions of an entire composite (size in the order of millimeters). Therefore scans of the surface (Figure S6b, d) and zoom-in of the cross section of a composite (Figure S6a,c,e) were done. Carbon peaks observed in both the spectra were predominantly from the alginate matrix, while peaks of Si and Fe were from AIP clay. The atomic weight percentage of N in the cross-section part (inner area) was higher than that of out surface. The result indicated that the elemental N was from Prussian blue nanoparticle. In addition, we measured SEM-EDX mapping data of the P-MSC composite (Figure S7). The SEM-EDX mapping images showed that PB nanoparticles were homogeneously embedded in the alginate/AIP clay composite regardless of the loading. A uniform distribution of C, O, Si, Fe, and N from alginate chain, AIP clay, and PB nanoparticles were observed in the composite. Additionally, the stability of materials in the colloidal system was investigated using zeta potential (Table S1). The zeta potential values of alginate, AIP clay, and Prussian blue in neutral pH were −33.34 mV, +41.36 mV, and −33.34 mV, respectively.

We estimated the PB content in the P-MSC composite using an ultraviolet-visible (UV-vis) spectroscopy. The release behavior of PB from P-MSC composite diluted to 3 and 4 times in pancreatin is shown in Figure S8a. The P-MSC composite was completely degraded by the pancreatin, leading to total release of PB^36^. The PB release was evaluated by monitoring the blue fluorescence of the P-MSC composite. The strong absorption peaks at 690 nm for both samples indicating the PB release. Thus, a standard calibration curve of absorbance vs. PB concentration (Figure S8b) was used to determine the concentration of PB released. The 3 and 4 times diluted composite samples showed a PB release concentration of 0.069 mM and 0.051 mM, respectively. According to the calibration data, we confirmed that about 4.755 mg of PB present in the 20 mg of P-MSC composite. Therefore, the P-MSC composite adsorbent contains 23.78% of PB content.

### Mechanical property and stability studies

**Figure 4:**
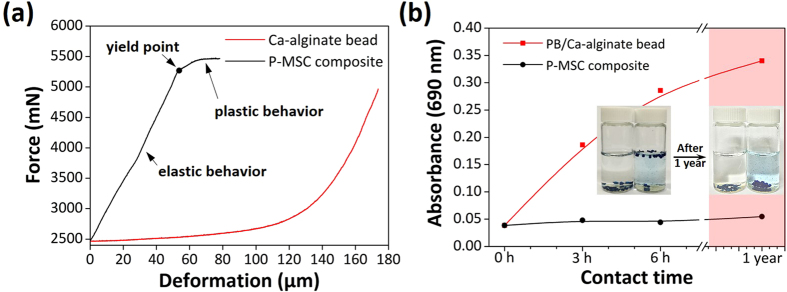
(**a**) Mechanical properties of Ca-alginate bead and P-MSC composite and (**b**) release behavior of PB from the PB/Ca-alginate bead and P-MSC composite (Fig. 4b inset shows the long-term study on stability of PB in the P-MSC composite solution during 1 year).

The mechanical properties of Ca-alginate and the P-MSC composite are shown in Fig. 4a. The principle of the measurement was to impose a force on the material by compression, and then to measure the corresponding force and the resulting deformation^37^. The Ca-alginate was soft, and it showed a strain hardening at a deformation value greater than 100 μm. The composite bore considerable loads up to a deformation value of 50 μm with a force of 5300 mN, and then it retained strain hardening. This behavior was attributed to the increase in the mechanical strength of the composite due the presence of the AIP clay. Additionally, nano-indentation is very suitable to check the mechanical properties of materials. Figure S9a illustrates the indentation depth vs. time curve recorded during a 100 second hold at a load of 45 mN. It was clear from the figure that gradual indenter penetrates into a bead surface under constant load. Instrumented indentation enables the determination of parameters for characterization of strength response. As a consequence, the apparent modulus and hardness decrease with time. An important question is that whether the deformations will be only delayed-reversible, or if also an irreversible creep will be present. As a result of indentation depth-time curve, there was no difference in the mechanical properties of Ca-alginate bead and PB/Ca-alginate bead. During the course of the instrumented indentation process, a record of the depth of penetration was made, and then the area of the indent was determined using the known geometry of the indentation tip. While indenting, various parameters such as load and depth of penetration can be measured. A record of these values can be plotted on a graph to create a load–indentation depth curves. The load–indentation depth curves for the Ca-alginate bead, PB/Ca-alginate bead, and P-MSC composite deflected to a central displacement of 5μm are shown (Figure S9b). These curves can be used to extract mechanical properties of the material^38^. The results showed that both Ca-alginate bead and PB/Ca-alginate bead surfaces were very smooth and weak as compared to P-MSC composite. Thus, the presence clay particles in the P-MSC composite was responsible for the increased mechanical property.

Adsorbent stability is an important factor because it is directly related to the adsorption capacity. Figure 4b shows the long term stability test of the PB/Ca-alginate and P-MSC composite measured by UV-vis spectroscopy. Ca-alginate is unstable in a physiological environment, and a rapid release of PB from alginate was observed. In contrast, the P-MSC composite was stable without the release of PB. This may be attributed to the fact that the AIP clay played a significant role in protecting the release of the PB nanoparticles from the P-MSC composite by cross-linking with alginate to improve the mechanical stability. In addition, the ionic interaction between the AIP clay, alginate, and the PB nanoparticles was another factor that influenced the PB release. Figure 4b inset shows the photograph of composite in solution after 1 year. There was no substantial release of PB from composite even after 1 year, indicating the excellent stability of the P-MSC composite. Thus, pure Ca-alginate could not be used in seawater as an adsorbent for the removal of radioactive cesium. Especially in a marine environment, Ca^2+^ ions are easily detached by the chelating agents and monovalent ions, such as Na^+^ and K^+^, found in seawater, which results in destabilization of the alginate matrix^20^. Figure S10 and Figure S11 shows the amount of PB released from PB/Ca-alginate bead, PB/Fe-alginate bead, and P-MSC composite in DI water and seawater. The results indicated that the PB/Ca-alginate bead released around 11 mM concentration of PB after 6 days. However, there was no substantial release of PB from the P-MSC composite. PB is composed of approximately 40% cyanide which is a toxicological concern^39^. The PB release data suggests that the P-MSC composite did not affect significantly the cyanide release. Thus, the toxicity resulting from cyanide release is not expected to be a medical concern. In this study, we successfully designed and synthesized a P-MSC composite with high stability and good mechanical property.

### Adsorption isotherms

The study of adsorption isotherm is an informative way to examine adsorbent performance. Two adsorption isotherm models, the Langmuir and Freundlich models, were used to investigate cesium adsorption. The Langmuir model^40^ is based on the assumption that all the active sites are equivalent and independent, and it indicates a monolayer adsorption process for cesium onto the uniformly adsorbent surface. The linear and nonlinear forms of the equation are written as:


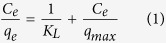



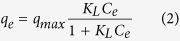


where *q*_*e*_ and *q*_*max*_ are the equilibrium adsorption capacity and monolayer maximum adsorption capacity (mg/g), respectively, and *K*_*L*_ is a constant related to the affinity between the adsorbent and the adsorbate (Figure S12a).

In comparison, the Freundlich adsorption isotherm model^41^ is considered to be an empirical equation that describes multi-layer adsorption with several types of adsorption sites on the surface of an adsorbent. The model consists of the following equations:


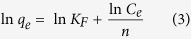



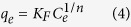


where *K*_*F*_ and *n* are the Freundlich constants relative to the multilayer adsorption capacity (Figure S12a).

**Figure 5:**
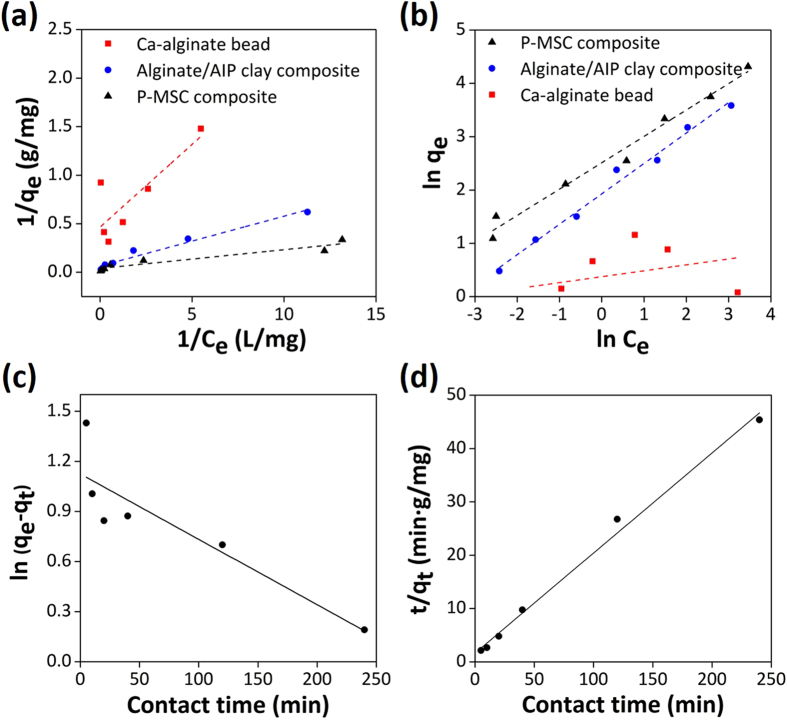
Adsorption isotherms and kinetics data. (**a**) Langmuir isotherm (**b**) Freundlich model, (**c**) pseudo-first-order, and (**d**) pseudo-second-order kinetics of the P-MSC composite.

In this present study, the adsorption capacity of the P-MSC composite as a function of cesium concentration was investigated by varying the initial concentration of cesium from 0.5 ppm to 50 ppm. The adsorption capacity increased rapidly with increasing cesium ion concentration when the initial concentration of cesium was less than 30 ppm, which may be attributed to the fact that sufficient active sites were available for the adsorption of cesium. At higher concentrations (above 30 ppm), competition for available adsorption sites could be decreased, resulting in a slower increase in the adsorption capacity. The experimental data were fitted to the Langmuir and Freundlich isotherm models. The linear regression correlation (*R*^*2*^) and constant values are listed in Table 1. For the Langmuir isotherm (Fig. 5a), a plot of 1/*C*_*e*_ against *1*/*q*_*e*_ produced a straight line with an *R*^*2*^ value of 0.95. For the Freundlich isotherm (Fig. 5b), the plot of ln *C*_*e*_ against ln *q*_*e*_ was used to evaluate the constants *K*_*F*_ and *n* from the slope and intercept, and the constant values were calculated to be 11.1 and 1.82, respectively. The isotherm showed a good fit with the Freundlich model, with an *R*^*2*^ value of 0.99. The *n* represents the heterogeneity factor, and it provides an indication of the favorability and capacity of the adsorption. The observed *n* value indicates the physical adsorption of cesium ions onto the composite. It was found that the composite possessed an impressive *q*_*m*_ of 108.06 mg/g for the cesium ion adsorption, which was higher than that of the Ca-alginate (2.25 mg/g) and alginate/AIP clay composite (48.48 mg/g). The result indicated that 1 unit of P-MSC composite (364.28 ± 17.37 μg) could adsorb approximately 39.4 μg of cesium.

### Adsorption kinetics

The adsorption kinetics were investigated *via* widely-used kinetic models (Figure S12b), such as pseudo first-order and second-order kinetics^42^,^43^. The adsorption mechanism and rate controlling steps, such as chemical reaction and mass transport, can be explained by first-order kinetics. The pseudo first-order rate equation can be expressed as:


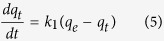


where *q*_*e*_ and *q*_*t*_ are the adsorption capacities (mg/g) at equilibrium and at time *t*, respectively, and *k*_*1*_ is the pseudo first-order rate constant (g/mg min). Upon integration and after applying boundary conditions *t* = 0 to *t* = t and q_*t*_ = 0 to *q*_*t*_ = *q*_*t*_, a simplified linear form of the rate equation can be obtained:


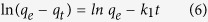


A plot of *ln (q*_*e*_*−q*_*t*_) versus *t* (Fig. 5c) showed a straight line with the *R*^*2*^ value of 0.75. From the slope and intercept of the straight line, the *q*_*e*_ and *k*_*1*_ values were calculated as 3.08 mg/g and 0.0039 g/mg min, respectively.

The second-order model is based on the assumption that the rate-limiting step may be chemisorption involving valence forces through the sharing of electrons between the adsorbent and the adsorbate as covalent forces. The second-order-rate equation can be described as follows:


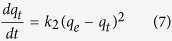



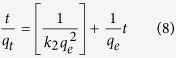


The *q*_*e*_ and *k*_*2*_ (second-order rate constant, g/mg min) values were calculated from the linear plot of *ln (q*_*e*_*−q*_*t*_) versus *t* (Fig. 5d) as 5.33 mg/g and 0.0214 g/mg min, respectively. The results revealed that the second-order model provided a better fit with an *R*^*2*^ value of 0.99, implying that the adsorption rate of the composite depends on the active sites rather than the concentration of cesium in the solution. The rate-limiting step was controlled by the chemical adsorption related to the exchange of protons between PB (present in the composite) and cesium^44^.

### Selectivity and adsorption mechanism

**Figure 6:**
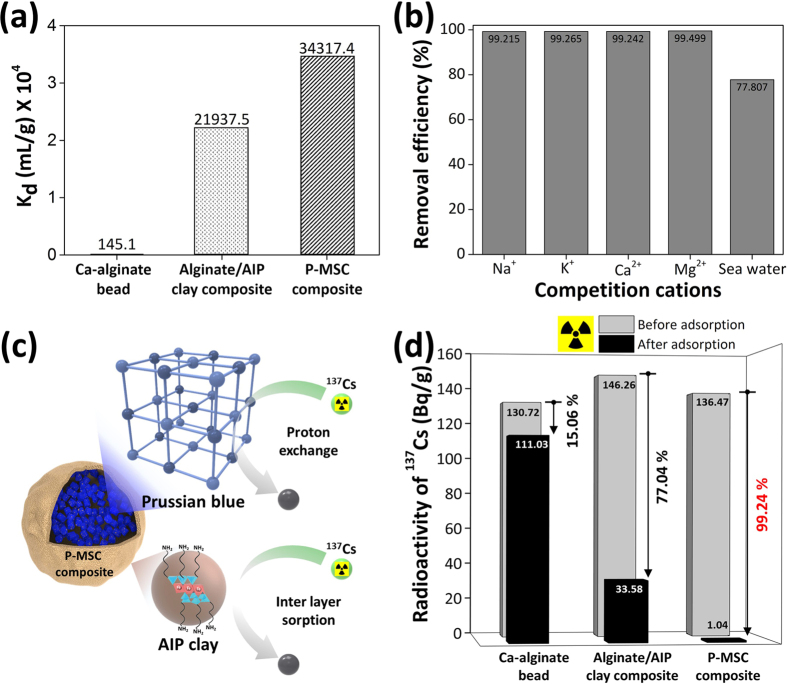
(**a**) Adsorption capacity values of the Ca-alginate, alginate/AIP clay composite, and P-MSC composite, (**b**) removal efficiency of cesium ion compared to competitor cations such as Na^+^, K^+^, Ca^2+^, Mg^2+^, and seawater (Cs concentration of 0.25 ppm or 1.88 × 10^−3^ mmol/L was initially spiked in all the samples, (**c**) adsorption mechanism, and (**d**) the removal efficiency of radioactive cesium using different adsorbents.

The cleanup of radioactive cesium is very difficult due to the lack of knowledge regarding the competition behaviors of the large number of mono- and divalent cations. The cesium adsorption capacity of Ca-alginate, the alginate/AIP clay composite, and the P-MSC composite was evaluated in terms of the distribution coefficient (*K*_*d*_). The *K*_*d*_ value was defined to evaluate the ability of the adsorbent to remove the cesium ions from the contaminated water:


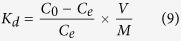


where *C*_*0*_ and *C*_*e*_ represent the initial and equilibrium concentration of cesium in solution, respectively, *V* is the volume of the cesium solution, and *M* is the mass of the adsorbent. At a cesium concentration of 0.2 ppm, the *K*_*d*_ values of Ca-alginate, alginate/AIP clay composite, and P-MSC composite were 145.1 mL/g, 21,937.5 mL/g, and 34,317.4 mL/g, respectively (Fig. 6a). The resulting composite had a *K*_*d*_ value that was 236-fold higher than that of Ca-alginate. As a comparison, we also synthesized Fe-alginate bead and PB/Fe-alginate bead, and evaluated the cesium adsorption capacity. The results are shown in Figure S13. The results showed that the Fe-alginate bead (80.9 mL/g) had a similar cesium adsorption capacity of Ca-alginate bead. The *K*_*d*_ value of PB contained Fe-alginate bead (2,982.4 mL/g) was lower than that of Alginate/AIP clay composite and P-MSC composite. The *K*_*d*_ value of P-MSC composite was 11.5-fold higher than that of the PB/Fe-alginate bead. This may be attributed to the high affinity of the PB nanoparticles towards cesium. The higher *K*_*d*_ value indicated a strong binding affinity, and this value was most meaningful at dilute concentrations of the adsorbate, which is similar to a real environment. Generally, a *K*_*d*_ value greater than 5,000 is considered to be good and a *K*_*d*_ value higher than 50,000 is considered to be excellent^45^. The selectivity of P-MSC composite for cesium (Fig. 6b) was evaluated by measuring the removal efficiency of cesium in the presence of competing cations such as Na^+^ (0.25 ppm), K^+^ (0.25 ppm), Ca^2+^ (0.25 ppm), Mg^2+^ (0.25 ppm) or real seawater contained an approximately sixteen thousand times higher concentration of competing cations than cesium concentration (0.25 ppm). A 0.25 ppm cesium was initially spiked in all the samples. The P-MSC composite showed excellent selectivity toward cesium ions because the concentration of cesium in any environment is significantly lower than the concentrations of co-existing cations, such as K^+^, Na^+^, Ca^2+^, and Mg^2+^. The competition ions are abundant in both fresh water and seawater. These results indicated that within the concentration of 9.4 × 10^−3^ mmol K^+^, Na^+^, Ca^2+^, and Mg^2+^, the cesium ion adsorption capacity of the P-MSC composite remained unchanged. The divalent cations had no effect or very little effect on the cesium ion adsorption. Although the removal efficiency of the cesium ions decreased at seawater, the P-MSC composite showed excellent selectivity toward the cesium ions even in the presence of competition cations that had a concentration that was approximately thousand-times higher than cesium (Figure S14). These results can be explained by the ability of PB to selectively adsorb hydrated cesium ions. The selectivity could be caused by the regular lattice spaces surrounded by the cyanide-bridged metals and the effect of the proton-exchange mechanism on the specific cesium ion adsorption (Fig. 6c)^30^,^46^. The adsorption ability of PB for alkali and alkaline earth metal ions increased in the order of Cs^+^ >> Na^+^, K^+^, Ca^2+^, and Mg^2+^.

### Decontamination of radioactive cesium

The efficiency percentage of the radioactive cesium removal using the P-MSC composite was calculated using the following equation:


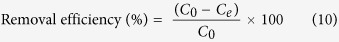


where *C*_*0*_ and *C*_*e*_ represent the initial and equilibrium concentrations of the cesium solution, respectively. Figure 6d shows the removal efficiency of radioactive cesium using Ca-alginate, the alginate/AIP composite, and the P-MSC composite. The adsorbents (1.0 mg/mL, each) were added to the radioactive cesium solutions (approximately 130 Bq/g) and agitated for 12 h. The adsorbents were then separated from the solutions by filtration through a syringe filter, after which the solutions were analyzed to determine the radioactive cesium concentration. The radioactive cesium removal efficiency reached 77.04% using the alginate/AIP composite, which was attributed to the large number of adsorption sites on the AIP clay. Further encapsulation of the PB nanoparticles in the adsorbent enhanced the removal efficiency up to 99.24%. Thus, the high removal efficiency further confirmed the potential application of the P-MSC composite for decontamination of water containing radioactive cesium.

## Conclusions

We successfully fabricated a 3D microporous composite by immobilization of AIP clay onto the PB/alginate matrix (i.e., ionic interaction between the AIP clay and the PB/alginate matrix) for selective adsorption of cesium ions from seawater. Pomegranate-like cage structured PB/alginate/AIP clay composite was confirmed by microscopy analysis. The adsorption results revealed that the composite showed a radioactive cesium removal efficiency of 99.24%. This may be attributed to the 3D porous network structure of the alginate/AIP clay and the good ion-exchange property of the PB. Experimental results demonstrated that the adsorption was in accordance with the Freundlish isotherm and consistent with second-order kinetics. It is anticipated that the P-MSC composite presents a new class of an environmentally-friendly adsorbent for the removal of radioactive cesium, which has been limited by natural clay or polymer-modified clays.

## Methods

### Materials

3-aminopropyltriethoxysilane (denoted APTES, ≥98%), iron (III) chloride, sodium alginate, Prussian blue (PB), and calcium chloride (CaCl_2_) were purchased from Sigma-Aldrich (USA) and used without further purification. The inactive cesium solution and radioactive cesium (^137^Cs) were obtained from Kanto Chemical Co. Inc. and Korea Atomic Energy Research Institute (KAERI), respectively. Seawater was obtained from the coastal sea area of Incheon, Korea.

### Preparation of AIP clay

The 3-Aminopropyl-functionalized iron phyllosilicate was synthesized as previously described^33^,^35^,^47^. In brief, the solution was prepared with FeCl_3_·6H_2_O (41.32 mmol, 8.4 g), which was dissolved in 200 mL of ethanol in a 500 mL beaker by stirring for 10 min. Then, 3-aminopropyltriethoxysilane (58.73 mmol, 13 mL) was added and adjusted to a molar ratio (FeCl_3_·6H_2_O to C_9_H_23_NO_3_Si) of 7:10. After mixing for about 5 min, a brown slurry was formed. To ensure sufficient equilibrium time for the AIP clay production, the reaction was continued overnight. The precipitated AIP clay was centrifuged at 6000 rpm for 10 min, and the pelleted material was washed twice with 200 mL ethanol by repeated centrifugation. The product was dried in an oven at 50 °C for 24 h. Prior to use, the AIP clay was powdered using a pestle and mortar. The unit structure of the clay ([H_2_N(CH_2_)_3_]_8_Si_8_Fe_6_O_12_(OH)_4_) contains a central octahedral brucite-like Fe(OH)_2_ in which the top and bottom are overlaid with tetrahedral silica^48^, followed by capping with vertical layers of flexible –(CH_2_)_3_NH_2_ groups^49^. Clay is easily prepared at room temperature *via* a sol–gel process using iron chloride and organotrialkoxysilane as precursors^50^. AIP clay has emerged as a new class of organic–inorganic layered materials that are derivatives of 2:1 trioctahedral iron phyllosilicates covalently bonded with aminopropyl moieties occupying the interlayer regions^51^. Supplementary Fig. S2 shows the structure of the AIP clay.

### Synthesis of Ca-alginate beads

Using a typical fabrication method, the CaCl_2_ flakes (0.2 g) were dissolved in deionized (DI) water (10 mL) by stirring for 1 h. Aqueous sodium alginate in DI water (10 mL, 2 wt%) was prepared separately, and then the alginate solution was added drop-wise into the CaCl_2_ solution. The hydrogel was shaken for 1 h, and then left to stand for 24 h. After collection, the hydrogel beads were immediately washed with DI water and freeze dried.

### Synthesis of the alginate/AIP clay composite

The alginate solution was prepared by dissolving 2 wt% sodium alginate in DI water. The AIP clay solution was prepared by dispersing 1.5 g of AIP clay nanoparticles in 30 mL DI water. Due to the intrinsic use of ferric ions in AIP clay synthesis, the clay was a brown color. The alginate solution was dropped into the AIP clay solution. The hydrogel was shaken for 1 h, and then it was left to stand for 24 h. After collection, the hydrogel beads were washed with DI water and freeze dried.

### Synthesis of the P-MSC composite

The P-MSC composite was fabricated using a similar procedure described for synthesis of the alginate/AIP clay composite. However, instead of the alginate solution, 50 μL of PB nanoparticles suspension (1 M) containing alginate solution (2%, 10 mL) was used.

### Characterization of the Ca-alginate beads, MSCs and P-MSCs

The SEM images were acquired using an S-4800SE microscope at an acceleration voltage of 15 kV. The FTIR spectra were recorded using a Jasco FT/IR-6600. The XRD patterns were collected using a Bruker D2 PHASER (Germany) diffractometer with Cu Kα radiation. The BET surface area and average pore diameter were obtained from the N_2_ adsorption/desorption isotherm using a fully automatic physisorption analyzer (ASAP 2020, Tristar). The XPS measurements were obtained using a Thermo Scientific, K–Alpha electron spectrometer with an Al X-ray source. UV-vis analysis were carried out using a V770 (JASCO) spectrophotometer. The mechanical properties were measured using micro-indenter measurements (Noise Is a Signal (NIS), customized). The inductively coupled plasma mass spectrometry (ICP-ms) measurements were carried out using a PerkinElmer ELAN6100. The radioactive cesium activity was measured using a High Purity Germanium (HPGe) detector (Canberra, USA).

### Zeta potential experiment

The zeta-potential values of the alginate, clay, and PB nanoparticle were measured in a Zeta-potential & Particle size Analyzer ELSZ (Ousuka, Japan). In most cases, colloidal particles possess a positive or negative electrostatic charge. As electrical fields are applied to the particle dispersion, the particles migrate in oppositely charged directions. As particles are irradiated in migration, scattering light causes Doppler shift depending on electrophoretic mobility. This method is called Laser Doppler Method. The essence of a classical micro-electrophoresis system is a cell with electrodes at either end to which a potential is applied. Particles move toward the electrode of opposite charge, and their velocity is measured. This velocity expressed per unit field strength is considered as its electrophoretic mobility of the particles. With this knowledge we can estimate the zeta-potential of the particles.

### The PB release behavior from the composite materials

The PB/Ca-alginate and P-MSC composites (each 10 mg) were separately immersed in DI water at room temperature for 6 h and shaken with a rotary shaker. The PB release behavior from the composite materials was evaluated by UV-vis spectroscopy at 690 nm.

### Adsorption isotherms

The adsorption isotherms were investigated based on batch experiments. Inactive cesium was used to study the adsorption behavior. The initial cesium concentration varied from 0.5 ppm to 50 ppm. A total of 10 mg of the adsorbent was added to 4 mL of the aqueous cesium solution. The test tube was then shaken at 60 rpm using a rotary shaker for 12 h. After reaching equilibrium, the adsorbent was separated by filtration, and the residual cesium concentration was analyzed using ICP-ms.

### Adsorption kinetics

For the kinetic studies, the sample was prepared by dispersing 20 mg of the P-MSC composite in 10 ppm of the cesium solution (10 ml) on a shaker at 200 rpm. The samples were taken at 5, 10, 20, 40, 90, 120, and 240 min.

### Selectivity experiment

Selectivity experiments were carried out using 5 mL of the cesium solution (0.25 ppm, 1.88 × 10^−3^ mmol/L) containing 10 mg of the P-MSC composite. After 12 h of agitation, the aqueous solution was removed and filtered through a syringe filter. The initial and residual cesium concentrations were analyzed using ICP-ms.

### Decontamination of radioactive cesium

The solution containing radioactive cesium was prepared by diluting a stock solution to approximately 130 Bq/g. The required amount of adsorbent (10 mg) was dispersed in 10 mL of the radioactive cesium solution, after which the vial was shaken for 12 h. Next, the aqueous solution was filtered through a syringe filter, and the adsorption capacity was measured using the HPGe detector (Canberra, USA).

## Additional Information

**How to cite this article**: Jang, S.-C. *et al*. Synergistically strengthened 3D micro-scavenger cage adsorbent for selective removal of radioactive cesium. *Sci. Rep.* **6**, 38384; doi: 10.1038/srep38384 (2016).

**Publisher's note:** Springer Nature remains neutral with regard to jurisdictional claims in published maps and institutional affiliations.

## Supplementary Material

Supporting Information

## Figures and Tables

**Table 1:** Langmuir and Freundlich model parameters for the adsorption of cesium ion onto Ca-alginate, alginate/AIP clay composite and P-MSC composite.

	Langmuir model			Freundlich model		
	*K*_*L*_ (L/mg)	*q*_*m*_ (mg/g)	*R*^*2*^	*K*_*F*_ (L/mg)	*n*	*R*^*2*^
Ca-alginate	4.53	2.25	0.39	1.56	9.91	0.005
Alginate/AIP clay composite	0.13	48.48	0.97	7.47	1.92	0.98
P-MSC composite	0.06	108.06	0.95	11.1	1.82	0.99
