# Supplementary material for: Synergistically strengthened 3D micro-scavenger cage adsorbent for selective removal of radioactive cesium
Source: Sci Rep. 2016 Dec 5;6:38384. doi: 10.1038/srep38384 (PMC5137142; doi:10.1038/srep38384)
Supplement: Supporting Information [file srep38384-s1.pdf]

Supplementary Information

Synergistically strengthened 3D micro-scavenger cage adsorbent for selective removal of radioactive cesium

Sung-Chan Jang[1,2,†], Sung-Min Kang[2,3,†], Yuvaraj Haldorai[4], Krishnan Giribabu[1], Go-Woon Lee[1,5], Young-Chul Lee[6], Moon Seop Hyun[7], Young-Kyu Han[4], Changhyun Roh[2,8,*] and Yun Suk Huh[1,*]

[1] Department of Biological Engineering, Biohybrid Systems Research Center (BSRC), Inha University, 100, Inha-ro, Incheon, 22212, Republic of Korea

[2] Biotechnology Research Division, Advanced Radiation Technology Institute (ARTI), Korea Atomic Energy Research Institute (KAERI), 29, Geumgu-gil, Jeongeup-si, Jeonbuk, 56212, Republic of Korea

[3] Department of Chemical Engineering, Chungnam National University, 99, Daehak-ro, Daejeon, 34134, Republic of Korea

[4] Department of Energy and Materials Engineering, Dongguk University, 30, Pildong-ro 1-gil, Seoul, 04620, Republic of Korea

[5] Quality Management Team, Korea Institute of Energy Research (KIER), 152, Gajeong-ro, Daejeon, 34129, Republic of Korea

[6] Department of BioNano Technology, Gachon University, 1342, Seongnam-daero, Seongnam-si, Gyeonggi-do, 13120, Republic of Korea

[7] Measurement & Analysisi Team, National Nanofab Center, 291, Daehak-ro, Daejeon, 34141, Republic of Korea

[8] Radiation Biotechnology and Applied Radioisotope Science, University of Science and Technology (UST), 217, Gajeong-ro, Daejeon, 34113, Republic of Korea

† These authors contributed equally to this work.

Correspondence and requests for materials should be addressed to C.R. (email: chroh@kaeri.re.kr) or Y.S.H. (email: yunsuk.huh@inha.ac.kr)

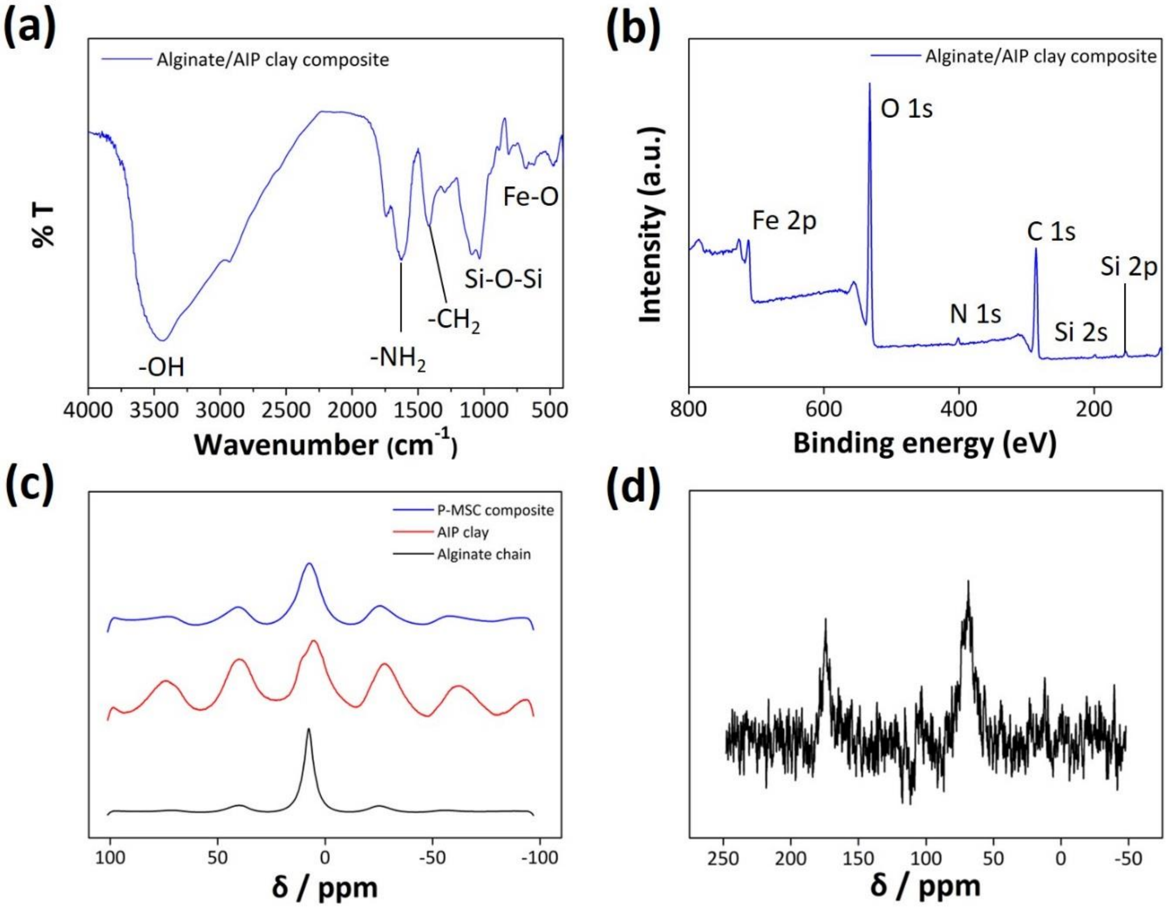

Figure S1. (a) FT-IR and (b) XPS spectra of alginate/AIP clay composite, (c) ^{1}H MAS NMR spectra of alginate, AIP clay, and P-MSC composite and (d) ^{13}C MAS NMR spectrum of P-MSC composite.

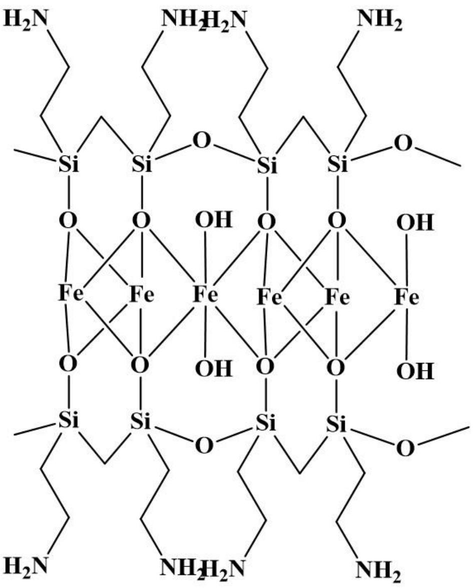

Figure S2. Chemical structure of AIP clay. AIP clay has the formula $[H_2N(CH_2)_3]_8Si_8Fe_6O_{12}(OH)_4$, and the approximate unit structure contains a central octahedral brucite sheet with the top and bottom being overlaid with tetrahedral silica followed by capping with vertical layers of flexible $-(CH_2)_3NH_2$ groups.

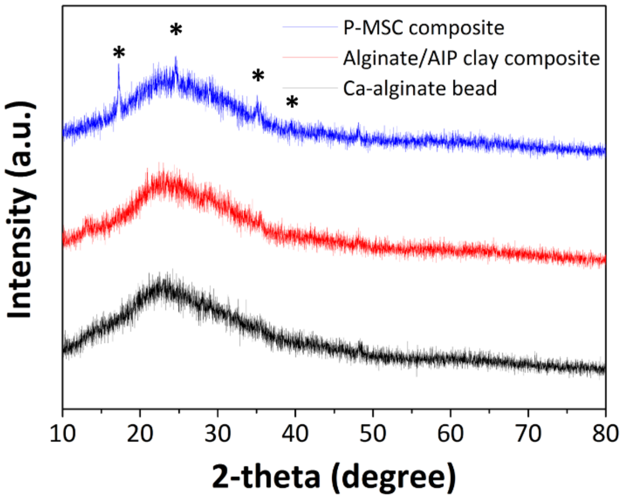

Figure S3. XRD data of the Ca-alginate bead, alginate/AIP clay composite, and P-MSC composite.

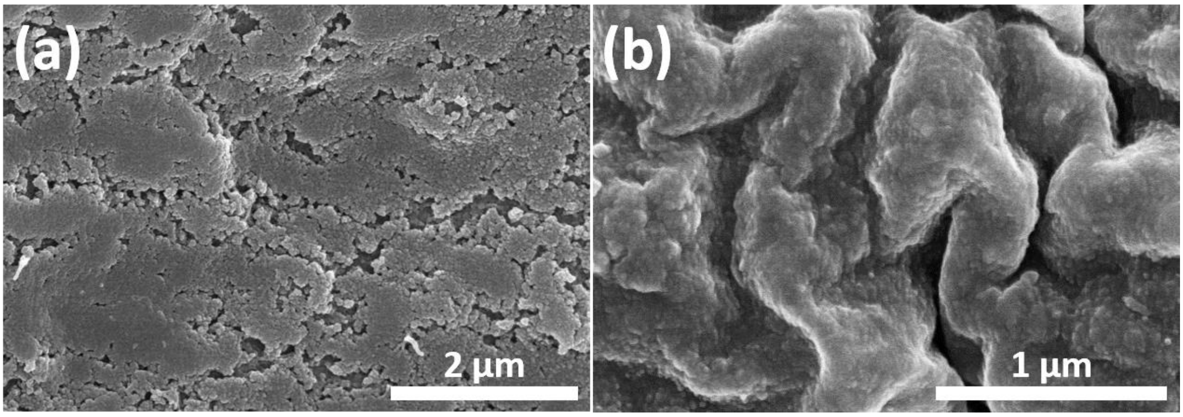

Figure S4. Surface SEM images of the (a) Ca-alginate bead and (b) P-MSC composite

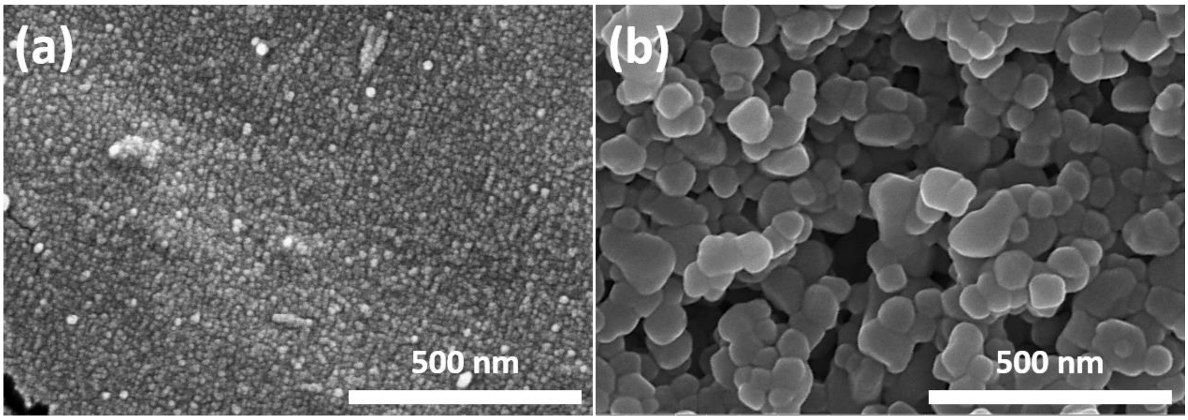

Figure S5. SEM images of the (a) AIP clay and (b) Prussian blue nanoparticles.

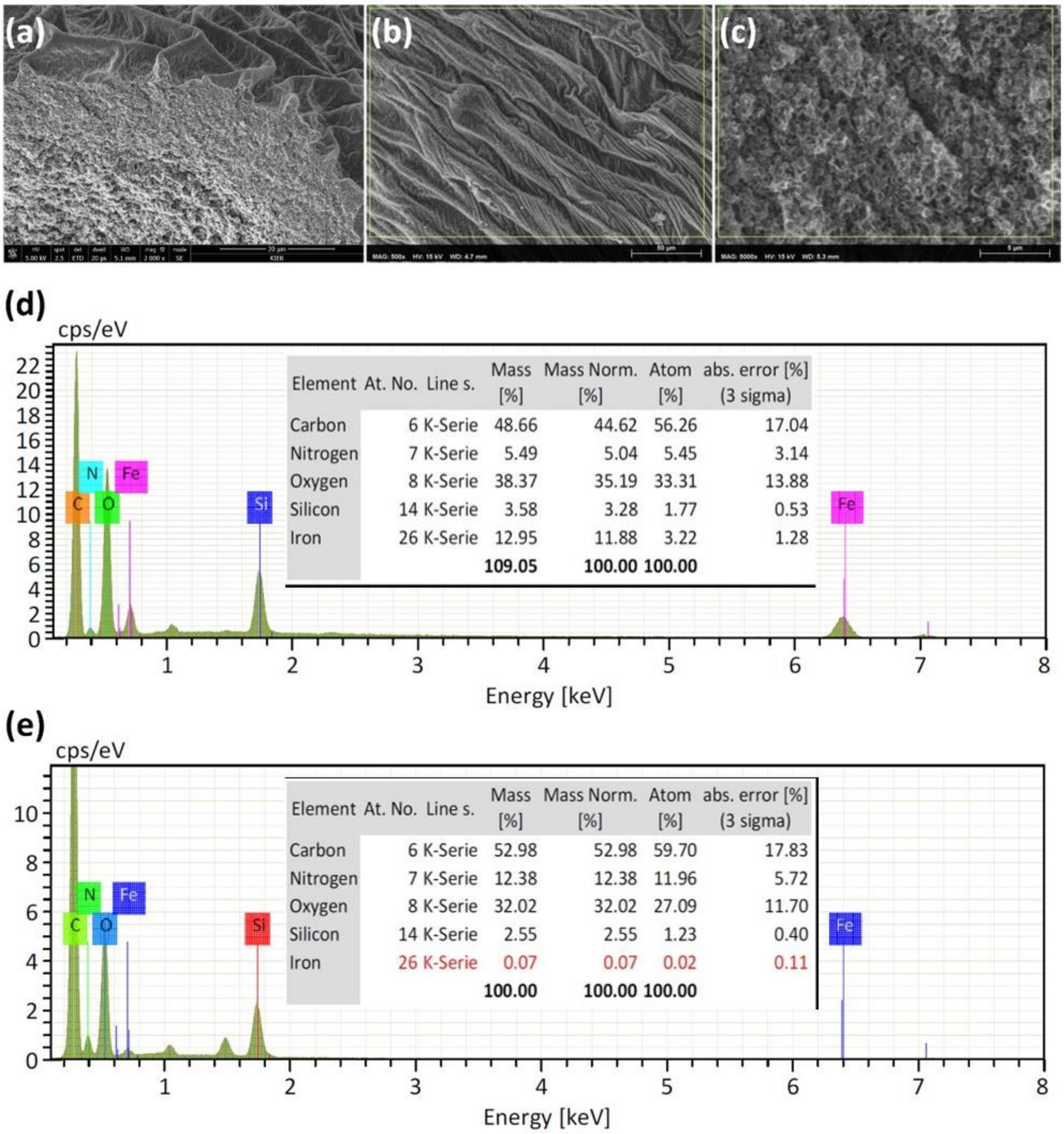

(d)

Element	At. No.	Line s.	Mass [%]	Mass Norm. [%]	Atom [%]	abs. error [%] (3 sigma)
Carbon	6	K-Serie	48.66	44.62	56.26	17.04
Nitrogen	7	K-Serie	5.49	5.04	5.45	3.14
Oxygen	8	K-Serie	38.37	35.19	33.31	13.88
Silicon	14	K-Serie	3.58	3.28	1.77	0.53
Iron	26	K-Serie	12.95	11.88	3.22	1.28
			**109.05**	**100.00**	**100.00**	

(e)

Element	At. No.	Line s.	Mass [%]	Mass Norm. [%]	Atom [%]	abs. error [%] (3 sigma)
Carbon	6	K-Serie	52.98	52.98	59.70	17.83
Nitrogen	7	K-Serie	12.38	12.38	11.96	5.72
Oxygen	8	K-Serie	32.02	32.02	27.09	11.70
Silicon	14	K-Serie	2.55	2.55	1.23	0.40
Iron	26	K-Serie	0.07	0.07	0.02	0.11
			**100.00**	**100.00**	**100.00**	

Figure S6. (a) SEM image of the cryo-fractured P-MSC composite, (b, c) the magnified images of the outer surface and inner structure of P-MSC composite, and (d, e) the corresponding EDX data.

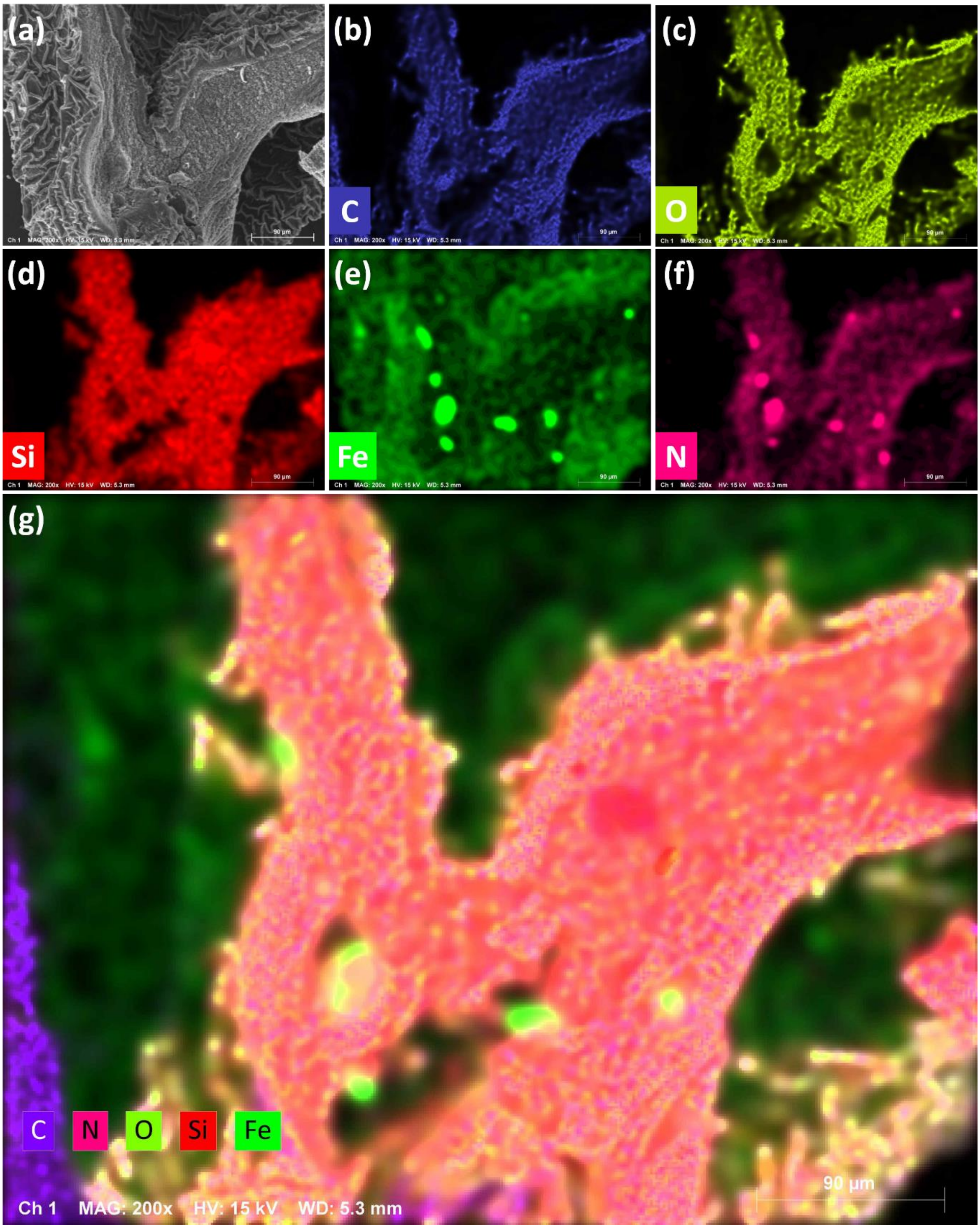

Figure S7. (a) Cross-section SEM image of P-MSC composite, (b-f) EDX mapping images, and (g) merged image.

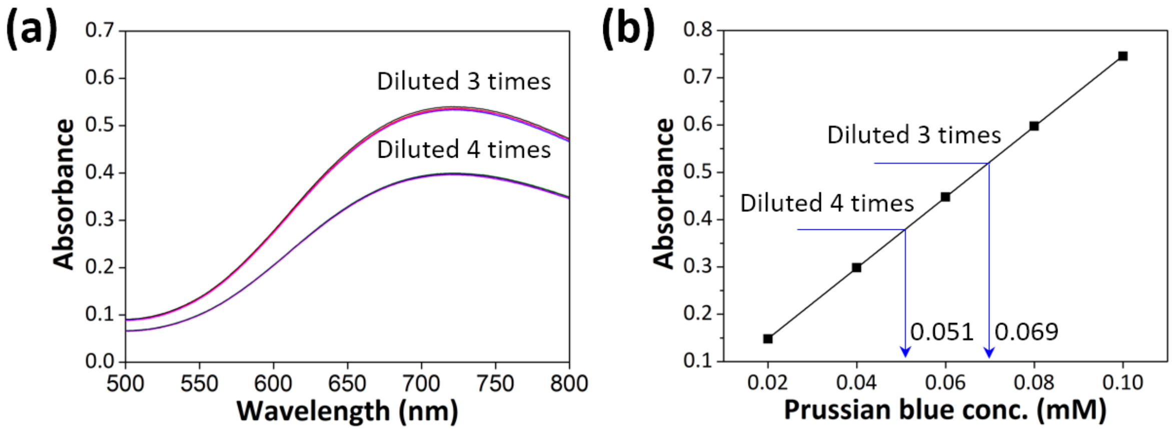

Figure S8. (a) The release behavior of PB from P-MSC composite diluted to 3 and 4 times and (b) calibration plot at 690 nm.

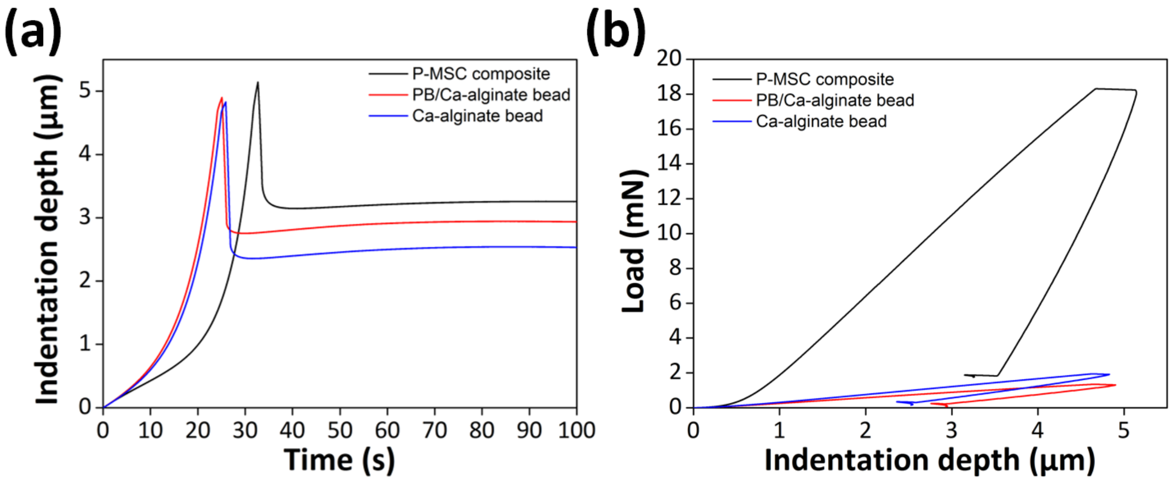

Figure S9. (a) Indentation depth vs. time curve recorded during a 100 second hold at a load of 45 mN and (b) a typical indentation load vs. indentation depth curve of Ca-alginate bead, PB/Ca-alginate bead, and P-MSC composite.

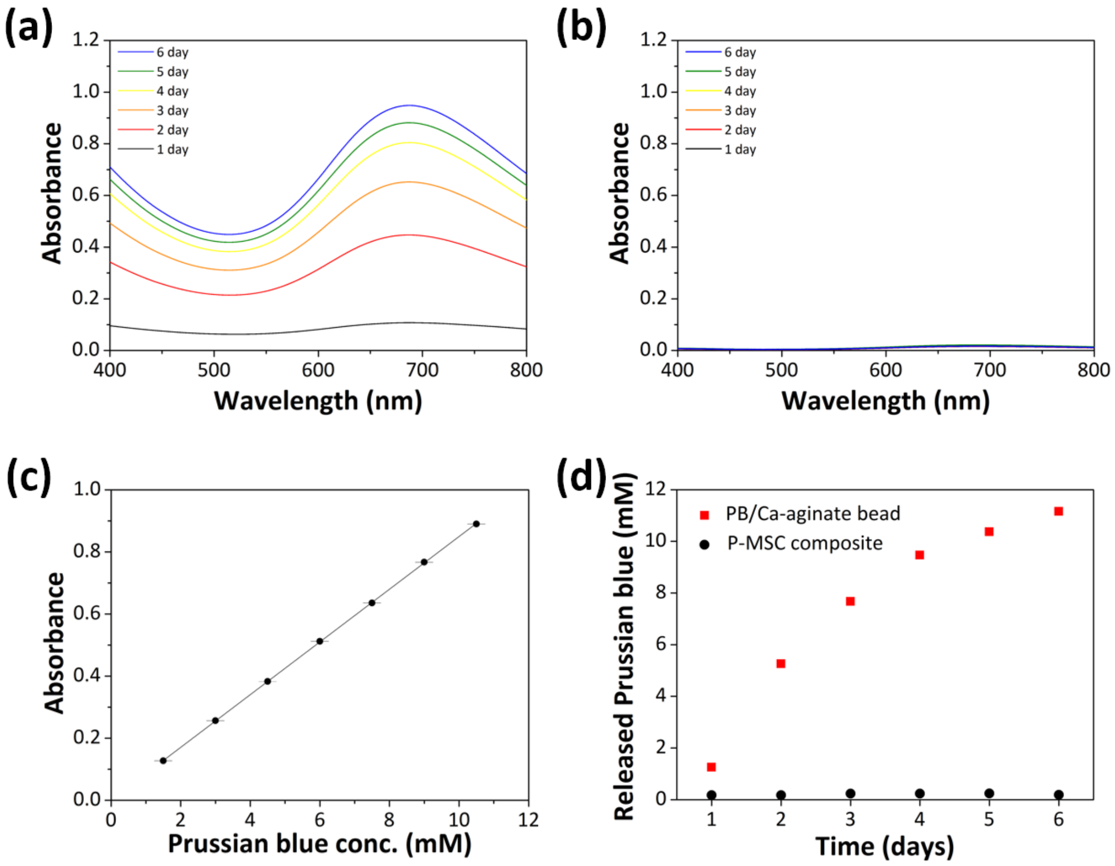

Figure S10. The release behavior of PB from (a) PB/Ca-alginate bead and (b) P-MSC composite was observed by UV-vis analysis during 6 days, (c) calibration curve, and (d) release profile.

Synthesis of Fe-alginate and PB/Fe-alginate beads. According to the reviewer's comment, we have synthesized Fe-alginate bead and PB/Fe-alginate bead, and evaluated their cesium adsorption capacity. In a typical experiment, 0.2 g of $FeCl_3 \cdot 6H_2O$ was dissolved in a 10 mL of deionized (DI) water under stirring for 1 h. Aqueous sodium alginate in DI water (10 mL, 2 wt%) was prepared separately, and then the alginate solution was added drop-wise to the above solution. The resulting mixture was shaken for 1 h, and then allowed to stand for 24 h. Finally, the hydrogel beads were washed with DI water and freeze dried.

The PB/Fe-alginate bead was fabricated using a similar procedure described for synthesis of the Fe-alginate bead. However, instead of the alginate solution, 50 μL of PB nanoparticles suspension (1 M) containing alginate solution (2 %, 10 mL) was used. And then the PB/alginate solution was added drop-wise into the $FeCl_3 \cdot 6H_2O$ solution. The hydrogel bead was shaken for 1 h, and then left to stand for 24 h. After collection, the hydrogel beads were immediately washed with DI water and freeze dried.

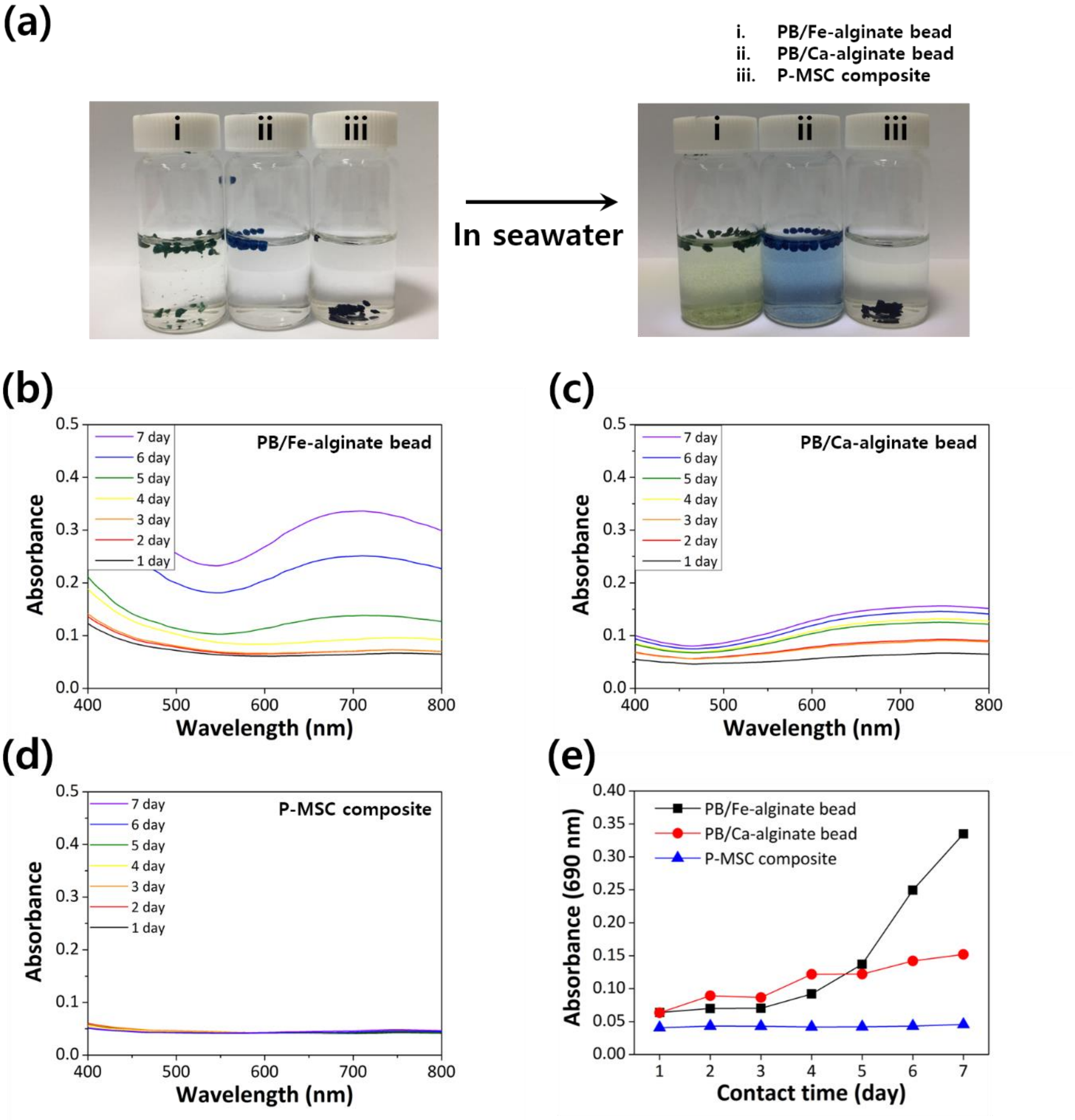

Figure S11. Release behavior of PB from PB/Fe-alginate bead, PB/Ca-alginate bead and P-MSC composite. (a) Photograph of the solution after 7 day. (b) PB/Fe-alginate bead, (c) PB/Ca-alginate bead and (d) P-MSC composite was observed by UV-vis analysis during 7 days. (e) Release profile of Prussian blue.

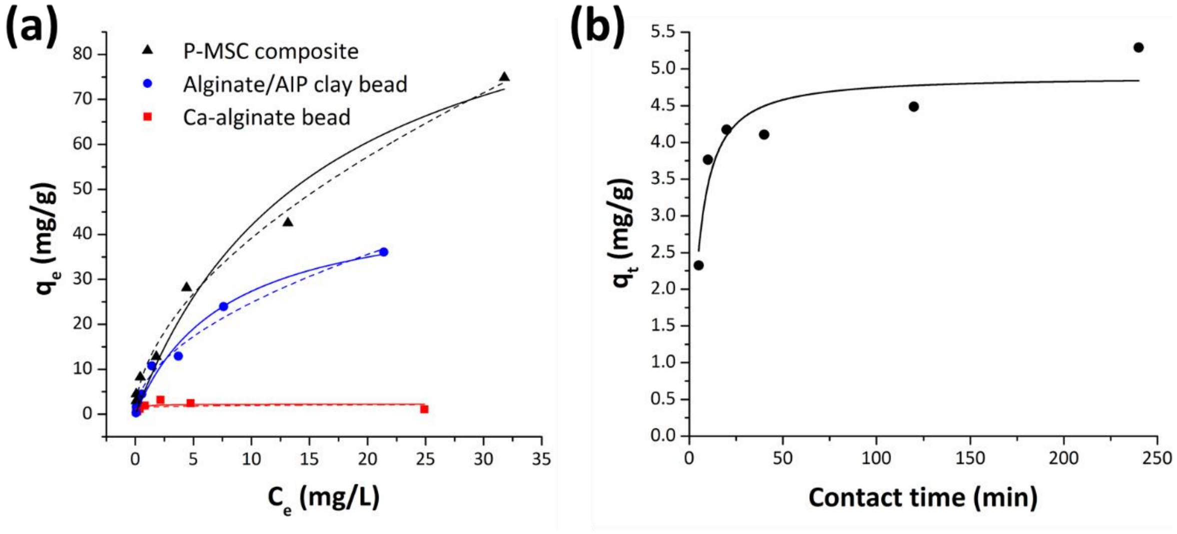

Figure S12. (a) Langmuir (solid line) and Freundlich (dotted line) adsorption isotherm models of Ca-alginate bead (■ red), Alginate/AIP clay composite, (● blue) and P-MSC composite (▲ black) and (b) adsorption kinetics of the P-MSC composite.

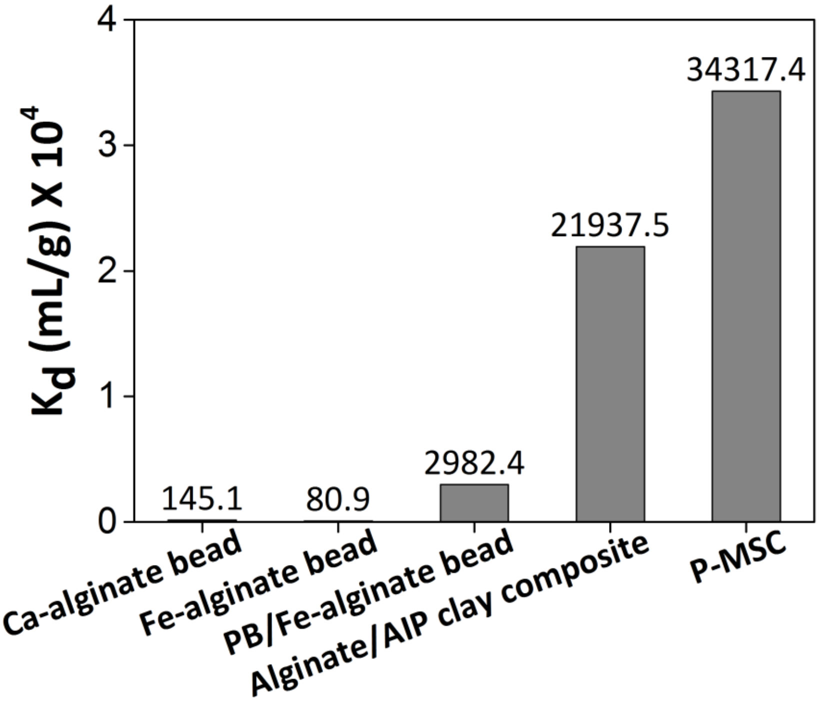

Figure S13. Adsorption capacity of cesium using the Ca-alginate bead, Fe-alginate bead, PB/Fe-alginate bead, alginate/AIP clay composite, and P-MSC composite as adsorbents.

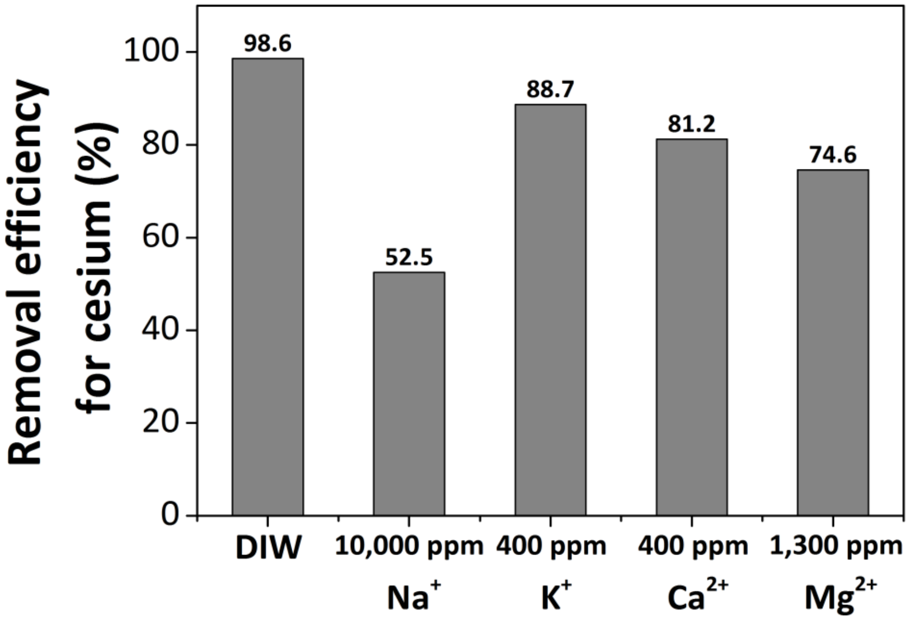

Figure S14. Removal efficiency of cesium ion compared to competitor cations such Na^{+}, K^{+}, Ca^{2+}, Mg^{2+}. An aqueous solution containing 0.24 ppm of cesium and high concentration of competing cations were prepared to simulate the contaminated water after a nuclear accident because the concentration of cesium diffused from nuclear fallout is significantly lower than the concentrations of cations in any environment.

Table S1. Zeta potential values of alginate, AIP clay, and Prussian blue.

A. Alginate

Zeta Potential	: -33.34	(mV)	Doppler shift	: 18.43	(Hz)
Mobility	: -2.600e-004	(cm^2/Vs)	Base Frequency	: 121.7	(Hz)
Conductivity	: 4.4363	(mS/cm)	Conversion Equation	: Smoulchowski	

B. AIP clay

Zeta Potential	: 41.36	(mV)	Doppler shift	: 23.38	(Hz)
Mobility	: 3.226e-004	(cm^2/Vs)	Base Frequency	: 120.0	(Hz)
Conductivity	: 3.5887	(mS/cm)	Conversion Equation	: Smoulchowski	

C. Prussian blue

Zeta Potential	: -33.84	(mV)	Doppler shift	: 21.60	(Hz)
Mobility	: -2.639e-004	(cm^2/Vs)	Base Frequency	: 121.5	(Hz)
Conductivity	: 0.0073	(mS/cm)	Conversion Equation	: Smoulchowski	
